# Managing hyperglycemia in patients with Cushing’s disease treated with pasireotide: medical expert recommendations

**DOI:** 10.1007/s11102-013-0483-3

**Published:** 2013-04-07

**Authors:** Annamaria Colao, Christophe De Block, Maria Sonia Gaztambide, Sudhesh Kumar, Jochen Seufert, Felipe F. Casanueva

**Affiliations:** 1 Dipartimento di Medicina Clinica e Chirurgia, Università di Napoli Federico II, Naples, Italy; 2 Department of Endocrinology, Diabetology and Metabolism, Antwerp University Hospital, Edegem, Belgium; 3 Department of Endocrinology, University Hospital Cruces (UPV-EHU), Vizcaya, Spain; 4 CIBERDEM (Centro de Investigación Biomédica en Red de Diabetes y Enfermedades Metabólicas Asociadas; ISCIII), Madrid, Spain; 5 University Hospital, Coventry, UK; 6 Division of Endocrinology and Diabetology, Department of Internal Medicine II, University Hospital of Freiburg, Freiburg, Germany; 7 Department of Medicine, Santiago de Compostela University, Santiago de Compostela, Spain; 8 CIBER Obesity and Nutrition, ISCIII, Madrid, Spain

**Keywords:** Cushing’s disease, Glucagon-like-peptide-1, Hyperglycemia, Pasireotide, Recommendations

## Abstract

To recommend an approach to monitoring and treating hyperglycemia in pasireotide-treated patients with Cushing’s disease, a severe clinical condition caused by a pituitary adenoma hypersecreting adrenocorticotropic hormone. Advisory Board meeting of ten European experts in pituitary disease and diabetes mellitus in Munich, Germany, on February 23, 2012, to obtain expert recommendations. Cushing’s disease presents a number of management challenges. Pasireotide, a novel agent for the treatment of Cushing’s disease with proven biochemical and clinical efficacy, improves outcomes and expands treatment options. Clinical trials have shown that the pasireotide adverse event profile is similar to that of other somatostatin analogs, except for a higher frequency of hyperglycemia. Mechanistic studies in healthy volunteers suggest that pasireotide-associated hyperglycemia is due to reduced secretion of glucagon-like peptide (GLP)-1, glucose-dependent insulinotropic polypeptide, and insulin; however, it is associated with intact postprandial glucagon secretion. Individual patients’ results demonstrate effective hyperglycemia management by following standard guidelines for the treatment of diabetes mellitus with individual adaptation to the specific underlying pathophysiology, i.e., preferential use of GLP-1 based-medications. Patients on pasireotide treatment should be monitored for changes in glucose metabolism and hyperglycemia. Diabetes mellitus should be managed by initiation of medical therapy with metformin and staged treatment intensification with a dipeptidyl peptidase-4 inhibitor, with a switch to a GLP-1 receptor agonist and initiation of insulin, as required, to achieve and maintain glycemic control. Further research into hyperglycemia following pasireotide treatment will help refine the optimal strategy in Cushing’s disease.

## Introduction

Cushing’s disease is a severe clinical condition caused by a pituitary adenoma hypersecreting adrenocorticotropic hormone (ACTH). The persistently high levels of ACTH lead to chronic hypersecretion of cortisol by the adrenal glands, which negatively affects many tissues and organs in the body. Cushing’s disease accounts for >70 % of the causes of endogenous chronic hypercortisolism, other causes include ectopic ACTH secretion by tumors, adrenal adenoma or carcinoma, macronodular or micronodular adrenal hyperplasia, and, more rarely, primary pigmented nodular adrenocortical disease and McCune–Albright syndrome [1]. Cushing’s disease is a rare disorder associated with substantial morbidity and mortality. It has an annual incidence of 1.2–2.4 cases per million population [2, 3] and a prevalence of 29.1 per million [3]. The effects of Cushing’s disease include metabolic and cardiovascular complications, osteoporosis and other bone alterations, kidney stones, autoimmune diseases, and susceptibility to opportunistic infections [4–15]. Mortality in patients with active Cushing’s disease is 4-times higher than in age- and sex-matched controls [3, 16].

The goals for treating Cushing’s disease are the reversal or amelioration of clinical symptoms by the normalization of cortisol levels to achieve minimal morbidity while incurring the fewest side effects possible. This can be realized by the removal of the tumor mass (while preserving pituitary function) and inhibition of tumor growth to achieve long-term control without recurrence. Several interventions exist to treat Cushing’s disease, including surgery, radiotherapy, and medical therapy. Although initial surgery is most frequently used as the first-line option, it often fails [1, 17]. Radiotherapy may be an option when initial surgery is unsuccessful. However, therapeutic effects are often delayed and become apparent sometimes only after many years [18]. Also, radiotherapy may have side effects including partial or complete pituitary deficiency [19]. Medical therapies can be useful in patients with persistent disease or recurrence after surgery, but many of the drugs applied have limited effects or are not approved for Cushing’s disease [17, 20]. Adrenostatic medical therapies, including ketoconazole, metyrapone, mitotane, and etomidate, may also be used for Cushing’s disease [17, 20]. Novel therapies directed to reduce cortisol levels or cortisol biological action such as 11-beta-hydroxylase inhibitors or blockers of the glucocorticoid receptor are also in development [21, 22].

Pasireotide (Signifor^®^; Novartis Pharma AG, Basel, Switzerland) is the first medical therapy approved in the European Union to treat adult patients with Cushing’s disease for whom surgery is not an option or for whom surgery has failed. Pasireotide is the only medical treatment targeting the pituitary adenoma and has demonstrated long-term effectiveness for the biochemical control and clinical improvement of patients with Cushing’s disease [23, 24]. It was approved based on the results of a pivotal phase III study [23]. In this study, 26.3 % of patients treated with pasireotide 900 μg twice daily and 14.6 % treated with pasireotide 600 μg twice daily achieved normal urinary free cortisol (UFC) levels at month 6 without dose escalation, and 50 of 103 patients had a substantial reduction (either normalization or at least reduction from baseline) in the UFC levels at 6 months [23]. In patients treated with pasireotide 900 μg twice daily and who had baseline UFC levels between 1.5- and 2-times the upper limit of normal, half achieved normalization of UFC [23]. Clinical signs and symptoms of Cushing’s disease improved in most patients in this study, including a significant reduction in body weight, systolic- and diastolic-blood pressure, and low-density lipoprotein cholesterol levels, with a reduction in mean UFC [23].

## Hyperglycemia secondary to hypercortisolism

**Fig. 1:**
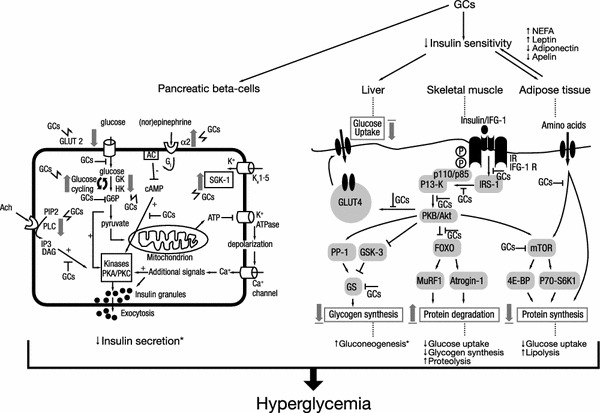
Pathophysiological mechanisms of glucocorticoid-induced diabetes. Adapted with permission from Refs. [25] © 2011 Elsevier; and [26] © 2009 John Wiley and Sons. *AC* adenyl cyclase, *Ach* acetylcholine, *ATP* adenosine triphosphate, *cAMP* cyclic adenosine monophosphate, *DAG* diacylglycerol, *4E-BP1* eIF4E-binding protein 1, *G6P* glucose-6-phosphatase, *G i* G-coupled inhibitory protein, *GC* glucocorticoid, *GK* glucokinase, *GLUT2* glucose transporter 2, *GLUT4* glucose transporter 4, *GS* glycogen synthase, *GSK-3* glycogen synthase kinase-3, *HK* hexokinase, *IFG-1* insulin-like growth factor-1, *IP3* inositol triphosphate, *Kv1·5* voltage-dependent K channel, *IR* insulin receptor, *IRS-1* insulin receptor substrate-1, *mTOR* mammalian target of rapamycin, *MuRF-1* muscle ring finger-1, *PI3-K* phosphatidylinositol-3 kinase, *PIP2* phosphatidylinositol biphosphate, *PKA* protein kinase A, *PKB* protein kinase B, *PKC* protein kinase C, *PLC* phospholipase C, *PP-1* protein phosphatase-1, *SGK-1* serum- and glucocorticoid inducible kinase-1, *S6K1* protein S6 kinase 1

Patients with Cushing’s disease are at a high risk of developing impaired glucose tolerance (IGT) and manifest diabetes secondary to hypercortisolism. A number of pathophysiological mechanisms underlie glucocorticoid-induced diabetes development in patients with Cushing’s disease (Fig. 1) [25, 26]. In general, chronic hypercortisolism blocks or impedes the action of insulin on peripheral tissues, such as liver, muscle and adipose tissue, leading to increased insulin resistance, and it partially inhibits insulin release by the pancreatic beta-cells. In particular, the glucocorticoid excess of patients with Cushing’s disease has a number of effects on glucose metabolism in hepatic tissue. These effects may be direct, such as stimulating gluconeogenesis through the induction of expression of essential enzymes [e.g., glucose transporter type 4, protein phosphatase 1, glycogen synthase kinase 3, and glycogen synthase (Fig. 1 provides an overview of the pathophysiological mechanisms of glucocorticoid-induced diabetes)] stimulating lipolysis and proteolysis with production of free fatty acids and amino acids which are substrates for gluconeogenesis, or the potentiation of hormone actions (especially glucagon) involved in glucose metabolism. Or they may be indirect, through the inhibition of insulin sensitivity by the depletion of glycogen storage in the liver [27–30]. The cortisol excess in patients with Cushing’s disease also leads to increased appetite and other central effects. Moreover, glucocorticoids induce pancreatic beta-cell dysfunction by inhibiting several signaling pathways and interfering at several steps in the insulin signaling cascade, particularly those that involve glucose cycling, glucose 6 phosphate, and protein kinase A and C activation (Fig. 1) [25, 26], all which may contribute to the risk of hyperglycemia secondary to hypercortisolism. Over time, patients are therefore likely to develop a disturbance of glucose homeostasis. In a recent study, the prevalence of IGT was 27 % in patients with Cushing’s disease and adrenal adenomas compared with 10 % in matched controls (*P* < 0.001) [31], which may develop further to manifest diabetes mellitus induced by the endocrine disease [32]. According to American Diabetes Association (ADA) classification, this type of diabetes can be classified under “Other specific types of diabetes due to other causes” or type 3 diabetes mellitus [32]. This diabetes should be managed in a similar way to type 2 diabetes mellitus (T2DM).

Generally, 40–45 % of patients with Cushing’s disease have been reported to develop diabetes and a further 10–30 % of patients have IGT [25, 33]. This compares with a phase II study which found that 5 % of patients with acromegaly treated with pasireotide developed diabetes, in addition to 7 % of patients showing increased blood glucose levels, and 5 % increased glycated hemoglobin (HbA_1C_) levels [34]. However, these figures may be an under-estimation of the true number of patients affected as specific testing of diabetes mellitus is not always performed in these patients.

## Hyperglycemia secondary to pasireotide treatment

Although the general safety profile of pasireotide in the pivotal study was consistent with first-generation somatostatin analogs (octreotide and lanreotide), hyperglycemia was observed in the majority of patients, with hyperglycemia-related adverse events reported in 72.8 % of patients [23]. Adverse events such as hyperglycemia and diabetes mellitus classified as grade 3 and 4 (according to the National Cancer Institute Common Terminology for Adverse Events version 3.0 [35]) occurred in up to 20 % of patients [23]. Levels of glucose and HbA_1C_ increased soon after the initiation of pasireotide treatment, and remained elevated throughout the treatment period in this study.

The pathophysiology of hyperglycemia secondary to pasireotide treatment, and methods for its management were investigated in two mechanistic studies in healthy volunteers [36]. These investigations have demonstrated that pasireotide administered over a short 8-day period decreases secretion of insulin by the beta-cell and intestinal secretion of glucagon-like peptide (GLP)-1 and glucose-dependent insulinotropic peptide, possibly through somatostatin receptor mediated effects on pancreatic beta-cells and entero-endocrine cells [36]. Hepatic and peripheral insulin sensitivity remains unchanged [36]. This diminished insulinotropic incretin effect may further contribute to the reduced insulin secretion that was observed. The studies suggest that in healthy volunteers dipeptidyl peptidase (DPP)-4 inhibitors (e.g., sitagliptin, vildagliptin, saxagliptin, linagliptin) and GLP-1 receptor agonists (e.g., liraglutide, exenatide) may be the most effective agents for reducing pasireotide-associated hyperglycemia [36].

On February 23, 2012, a group of ten European experts in the field of pituitary diseases and diabetes mellitus met at an Advisory Board meeting in Munich, Germany, to propose an approach to the management of hyperglycemia in patients with Cushing’s disease treated with pasireotide. This paper provides medical expert recommendations on the monitoring and treatment of hyperglycemia in patients with Cushing’s disease. The recent approval of pasireotide as a medical therapy for patients with persistent or recurrent Cushing’s disease for whom surgery is not an option or for whom surgery has failed renders these recommendations especially timely.

## Methods

These recommendations were prepared from the outcomes of the Advisory Board meeting and this article presents a summary of the discussions of the experts.

## Results

### Expert recommendations

#### For monitoring patients with Cushing’s disease treated with pasireotide

The results of the pivotal phase III study of pasireotide in patients with Cushing’s disease demonstrated that the majority of patients treated with pasireotide will develop hyperglycemia [23]. Therefore, all patients treated with pasireotide should be monitored for the development of IGT or manifest diabetes mellitus. Current definitions used by the ADA for the diagnosis of manifest diabetes are an HbA_1C_ level of ≥6.5 %, a fasting plasma glucose level of ≥126 mg/dL (7.0 mmol/L), a 2-h plasma glucose level of ≥200 mg/dL (11.1 mmol/L) during an oral glucose tolerance test; or in patients with classic symptoms of hyperglycemia or hyperglycemic crisis, a random plasma glucose level of ≥200 mg/dL (11.1 mmol/L) [32].

Patients with normal glucose metabolism prior to pasireotide therapy should self-monitor their fasting and postprandial glucose levels by taking several blood glucose measurements per day (e.g., before and 2 h after breakfast, lunch, and dinner). They should do so twice in the first week and once weekly thereafter. Patients with impaired fasting glycemia (IFG) and/or IGT, and patients with diabetes under treatment, must be closely monitored on a daily basis at the start of treatment with pasireotide and, if needed, treatment shall be adjusted or changed in order to control the new level of glycemia. Their treating physician should follow-up closely (e.g., after 1, 2, and 4 weeks) to assess the emergence of any hyperglycemia and initiate appropriate therapy.

#### For treating hyperglycemia secondary to pasireotide therapy in patients with Cushing’s disease

**Fig. 2:**
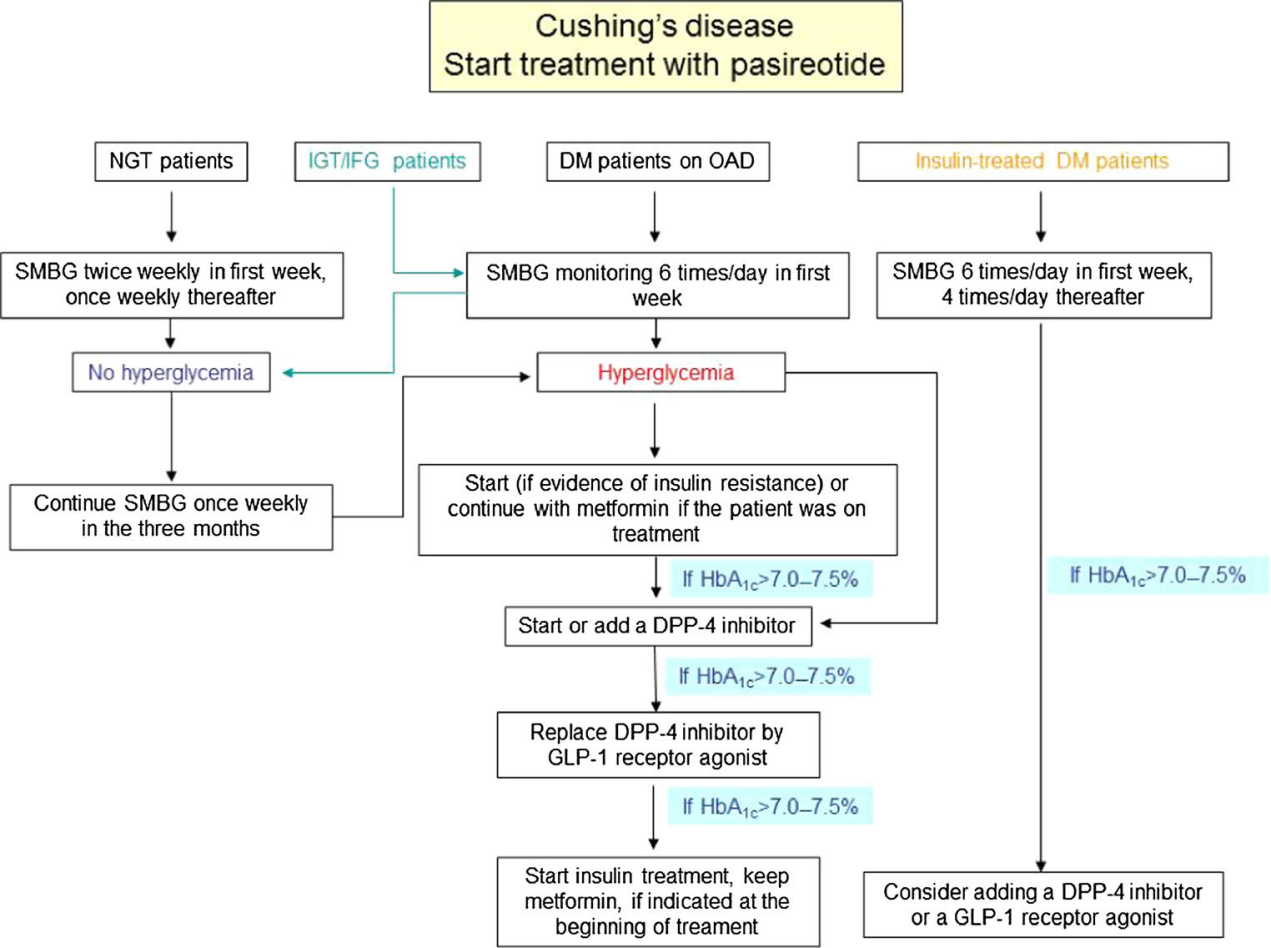
Recommendations on the monitoring and treatment of hyperglycemia in patients with Cushing’s disease treated with pasireotide. In case patients fail to control glucose levels with GLP-1 agonists, as stated in the text, we suggest moving to insulin. *DDP-4* dipeptidyl peptidase-4, *DM* diabetes mellitus, *GLP-1* glucagon-like peptide-1, *HbA*_*1C*_ glycated hemoglobin, *IFG* impaired fasting glycemia, *IGT* impaired glucose tolerance, *NGT* normal glucose tolerance, *OAD* oral antidiabetic drugs, *SMBG* self-monitoring of blood glucose

Treatment for patients with Cushing’s disease, whether they present with IFG and/or IGT or diabetes, or develop hyperglycemia secondary to treatment with pasireotide, should be based on the currently recommended treatment algorithms for T2DM. Thus, individually tailored glycemic targets of HbA_1C_ level of <7.0–7.5 % (<53–58 mmol/mol) are appropriate unless the treating clinician perceives a risk due to hypoglycemia, or if the patient is unlikely to survive long enough to be at risk of complications. Medical treatment should include dietary modification, exercise, and education. In patients with evidence of insulin resistance, medication should be initiated with metformin as the first-line therapy unless contraindicated or not well tolerated (Fig. 2) [33, 37].

As pasireotide-related hyperglycemia is associated with both decreased insulin secretion and a reduced incretin response [34], anti-hyperglycemic treatment in patients with Cushing’s disease treated with pasireotide should preferentially address these two pathophysiological mechanisms. If control of glycemia is not achieved or maintained with metformin alone, combination therapy with agents targeting the incretin-pathway is recommended [36]. Based on the preliminary data mentioned above [36], it is suggested that in hyperglycemia secondary to pasireotide treatment, metformin in combination with a GLP-1-based treatment option may be specifically advantageous (Fig. 2). Therefore, in a first step combination, therapy with a DPP-4 inhibitor may be established. In addition, to target the specific pathophysiology of hyperglycemia secondary to pasireotide treatment, this combination provides the specific advantages of a low hypoglycemic potential together with a neutral effect on body weight. Although combination therapies of metformin with sulfonylureas and/or pioglitazone may also be possible theoretically, these are not recommended as sulphonylureas stimulate insulin secretion in a glucose-independent manner and, therefore, are associated with a substantial risk of hypoglycemia and weight gain. Furthermore, treatment failure due to a decline of pancreatic beta-cell function may occur more rapidly with sulphonylurea therapy. Pioglitazone primarily targets insulin resistance and does not, therefore, represent a pathophysiologically-oriented treatment option for hyperglycemia secondary to pasireotide therapy. Moreover, pioglitazone treatment has been associated with an increase in body weight, fluid retention, and bone fractures—all side effects that are most unwanted in Cushing’s disease patients [38].

If glycemic targets are not reached with the metformin plus DDP-4 inhibitor combination, the DPP-4 inhibitor may be replaced by a GLP-1 receptor agonist. These GLP-1 analogs have demonstrated an HbA_1C_-lowering effect that is superior to that of DPP-4 inhibitors without increase of hypoglycemia risk. In addition, they provide the potential to reduce body weight, which is desirable in Cushing’s disease. If hyperglycemia secondary to pasireotide treatment in patients with Cushing’s disease remains uncontrolled by these combinations, establishing insulin therapy together with maintained metformin treatment may be necessary. In these cases, initial combination therapy of metformin with a once-daily application of a long-acting basal insulin analog (either glargine or detemir since they have similar efficacy on glucose target [39]) targeting levels of fasting plasma glucose considered acceptable by the treating physician, may be the first option. If the individual HbA_1C_ targets are not met or the postprandial glucose levels are high with this basal insulin-supported anti-diabetic therapy, then prandial insulin therapy also has to be finally established.

These recommendations are supported by similar proposals on the management of hyperglycemia in Cushing’s disease developed from a review of the existing literature [40]. Reznik et al. suggest that patients should be tested for impaired glucose regulation before initiating treatment with pasireotide. They should be monitored for glycemic control on initiation of pasireotide and also as part of regular follow-ups and, if glycemic control deteriorates during pasireotide therapy, they should be treated with antidiabetic therapy as appropriate [40].

## Further research

A study of patients with Cushing’s disease who perform regular monitoring of blood glucose would provide information to help stratify patients by risk of hyperglycemia and assist the development of specific guidelines for managing this risk in the future. At this point, however, it seems prudent to apply a similar clinical risk estimation for the development of glycemic disorders in patients with Cushing’s disease as is standard in patients at-risk for classical T2DM. This involves assessing features of the metabolic syndrome, such as visceral obesity, concomitant dyslipoproteinemia and hypertension, and familial background for metabolic disease. Whether validated diabetes risk questionnaires such as the ones developed from the Study to Prevent Non-Insulin Dependent Diabetes Mellitus (STOP-NIDDM) and the Finnish Cardiovascular Risk Factor (FINDRISK) [41, 42], may help to identify patients at risk in the setting of Cushing’s disease is unknown.

Significant progress in understanding the fundamental pathophysiology of hyperglycemia secondary to pasireotide treatment has been made since the phenomenon was first recognized. However, further research on how pasireotide influences insulin biosynthesis and secretion, and insulin sensitivity in patients with Cushing’s disease will help to define the optimum treatment strategies for hyperglycemia in this difficult to manage condition.

Although metformin followed by the addition of a DPP-4 inhibitor, with a switch from the DPP-4 inhibitor to a GLP-1 receptor agonist or insulin therapy as required to maintain glycemic targets, is suggested as a suitable treatment of hyperglycemia secondary to pasireotide therapy, the efficacy of anti-hyperglycemic regimens needs to be further explored in a clinical trial in patients with Cushing’s disease.

## Summary

Patients with Cushing’s disease have a high risk of hyperglycemia due to the long-term effect of hypercortisolism, and emergence or worsening of hyperglycemia is frequently observed secondary to pasireotide treatment. However, mechanistic studies and individual observations in patients with Cushing’s disease suggest that pasireotide-related hyperglycemia responds to anti-diabetic agents following similar protocols recommended for the treatment of T2DM, with a specific focus on GLP-1-based medications. More research is necessary to confirm these results and to refine the recommendations for diagnosis and treatment of hyperglycemia in patients with Cushing’s disease in order to optimize their management.
